# Supplementary material for: Flat-band localization and self-collimation of light in photonic crystals
Source: Sci Rep. 2019 Feb 27;9:2862. doi: 10.1038/s41598-019-39471-0 (PMC6393537; doi:10.1038/s41598-019-39471-0)
Supplement: Supplementary file 1 — Supplementary Information: Flat-band localization and self-collimation of light in photonic crystals [file 41598_2019_39471_MOESM1_ESM.pdf]

Supplementary Information: Flat-band localization and self-collimation of light in photonic crystals

Nojoon Myoung,[1] Hee Chul Park,[2] Ajith Ramachandran,[2,3]
Elefterios Lidorikis,[4] and Jung-Wan Ryu[2,*]

[1]*Department of Physics Education, Chosun University, Gwangju 61452, Republic of Korea*
[2]*Center for Theoretical Physics of Complex Systems, Institute for Basic Science (IBS), Daejeon 34126, Republic of Korea*
[3]*Department of Physics, Indian Institute of Science Education and Research (IISER) Bhopal, Madhya Pradesh 462006, India*
[4]*Department of Materials Science and Engineering, University of Ioannina, Ioannina 45110, Greece*

[*] corresponding to jungwanryu@gmail.com

I. CONVERGENCE OF PHOTONIC BANDS: EFFECTS OF RESOLUTION

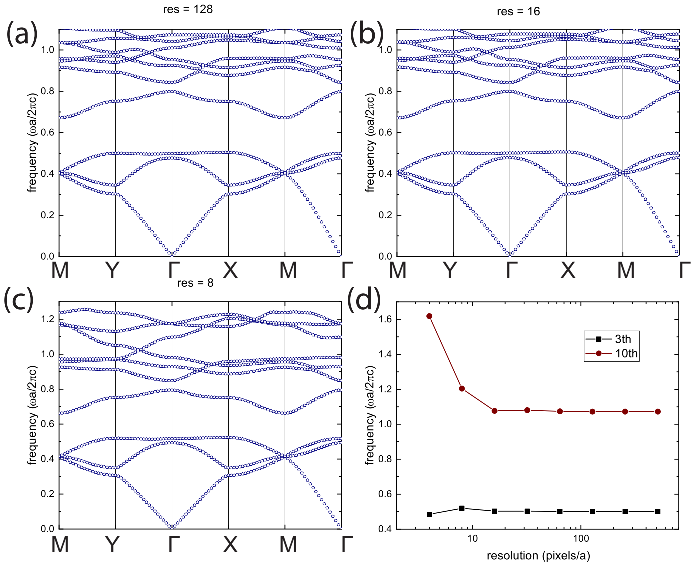

FIG. 1. Band structures of 2D side-coupled tunable diamond lattices with resolution (a) 128, (b) 16, (c) 8 pixels per a. (d) Frequencies of the 3rd and 10th bands for momentum at the center of $\Gamma \rightarrow X$ line, as functions of resolution.

In principle, numerical computations of photonic bands are based on finite-difference in space domain, which is related to the accuracy in terms of frequency. Numerical errors due to the spatial resolution may influence numerical results, if simulation sets are not adequately made. In this study, it is an important issue how highly the resolution is set, because small fluctuations by numerical errors may largely affect analysis of the flat band (FB). Our resolution in use for the calculations is 32×32 pixels in a unit cell. As shown in Fig. 1, our resolution is high enough to secure the convergence of the FB frequency. Therefore, our finding of the FBs is not a consequence of numerical errors due to low resolution.

II. EFFECTS OF NEXT-NEAREST-NEIGHBOR INTERACTION ON PHOTONIC BANDS

The photonic crystal is well defined by a tight-binding model with higher-order hopping. Here, we investigate band structures for 2D lattice models, by varying the hopping order. The tight-binding model with the higher-order hoppings reflects more realistic lattices like

photonic crystals where complicated interactions of electromagnetic fields exist.

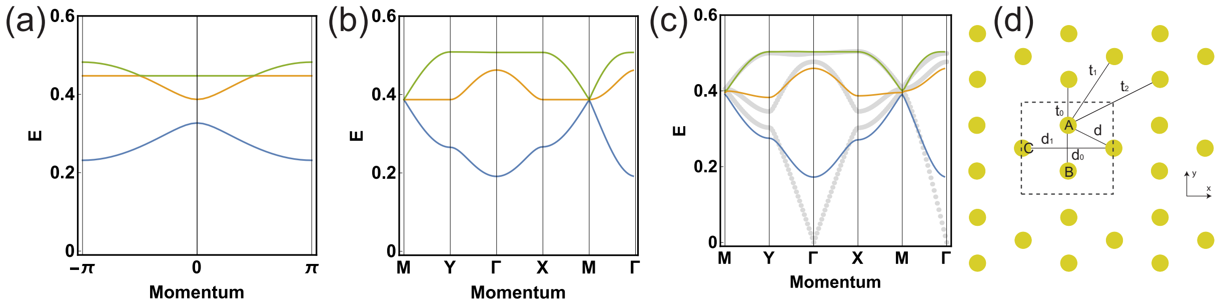

FIG. 2. Band structures from tight-binding models of (a) 1D tunable diamond chain, and 2D side-coupled tunable diamond lattices (b) with the side-couple hoppings and (c) with inter-cell hoppings. Numerical results from FDTD simulations are also plotted in (c) with gray dots. Momentum and energies E are given as dimensionless quantities, i.e., $ka/2\pi c$ and $\omega a/2\pi c$, respectively. (d) Hopping terms in use for the tight-binding calculations. Dashed rectangle represents the unit cell, containing three lattice sites in total.

Let us start with the simplest case, a 1D tunable diamond chain. The corresponding equation reads $\varepsilon\phi_n = H_0\phi_n + H_1^\dagger\phi_{n+1} + H_1\phi_{n-1}$, where the matrix elements are

$$H_0 = \begin{pmatrix} \epsilon_a & -d_0 & -d \\ -d_0 & \epsilon_b & -d \\ -d & -d & \epsilon_c \end{pmatrix}, \quad H_1 = \begin{pmatrix} 0 & 0 & 0 \\ 0 & 0 & 0 \\ -d & -d & 0 \end{pmatrix}. \tag{1}$$

The translation symmetry along the chain guarantees that the Hamiltonian is expressed through Bloch theorem as following,

$$H_{1D}\left(k_x\right) = \begin{pmatrix} \epsilon_a & -d_0 & -d\left(1+e^{ik_x}\right) \\ -d_0 & \epsilon_b & -d\left(1+e^{ik_x}\right) \\ -d\left(1+e^{-ik_x}\right) & -d\left(1+e^{-ik_x}\right) & \epsilon_c \end{pmatrix}, \tag{2}$$

where k_x is dimensionless with lattice constant. When we consider the case of uniform on-site potential, the Hamiltonian is reduced with zero diagonal elements, and the eigenvalues are obtained as $-d_0/2 \pm \sqrt{(d_0/2)^2 + (4d\cos k_x/2)^2}$ and d_0: the former and latter represent dispersive and FB in momentum space as depicted in Fig. 2(a).

In case of 2D, we can consider higher-order hopping terms up to the forth nearest-neighbor hoppings. Here, t_0 is hopping integral, describing the side coupling between

the adjacent 1D tunable diamond chains. We assign the higher-order hopping integrals d_1, t_1, t_2 as denoted in Fig. 2(d). Let us define that d, d_0 are intra-cell hopping integrals and d_1, t_0, t_1, t_2 are inter-cell hopping integrals, respectively. The corresponding equation reads $\varepsilon\phi_{n,m} = H_0\phi_{n,m} + H_{10}^{\dagger}\phi_{n+1,m} + H_{10}\phi_{n-1,m} + H_{01}^{\dagger}\phi_{n,m+1} + H_{01}\phi_{n,m-1} + H_{diag}^{\dagger}\phi_{n+1,m+1} + H_{diag}\phi_{n-1,m-1} + H_{off}^{\dagger}\phi_{n+1,m-1} + H_{off}\phi_{n-1,m+1}$, where the matrix elements are

$$H_0 = \begin{pmatrix} \epsilon_a & -d_0 & -d \\ -d_0 & \epsilon_b & -d \\ -d & -d & \epsilon_c \end{pmatrix}, \; H_{10} = \begin{pmatrix} -d_1 & 0 & 0 \\ 0 & -d_1 & 0 \\ -d & -d & -d_1 \end{pmatrix}, \; H_{01} = \begin{pmatrix} -t_2 & 0 & 0 \\ -t_0 & -t_2 & -t_1 \\ -t_1 & 0 & -t_2 \end{pmatrix},$$
$$H_{diag} = \begin{pmatrix} 0 & 0 & 0 \\ -t_2 & 0 & 0 \\ -t_1 & 0 & 0 \end{pmatrix}, \; H_{off} = \begin{pmatrix} 0 & -t_2 & 0 \\ 0 & 0 & 0 \\ 0 & -t_1 & 0 \end{pmatrix}. \tag{3}$$

In this case, the Bloch Hamiltonian is written by

$$H_{2D}\left(\vec{k}\right) = \begin{pmatrix} \epsilon_a - 2d_1\cos k_x - 2t_2\cos k_y & -d_0 - e^{ik_y}\left(t_0 + 2t_2\cos k_x\right) & -\left(1+e^{ik_x}\right)\left(d + e^{ik_y}t_1\right) \\ -d_0 - e^{-ik_y}\left(t_0 + 2t_2\cos k_x\right) & \epsilon_b - 2d_1\cos k_x - 2t_2\cos k_y & -\left(1+e^{ik_x}\right)\left(d + e^{-ik_y}t_1\right) \\ -\left(1+e^{-ik_x}\right)\left(d + e^{-ik_y}t_1\right) & -\left(1+e^{-ik_x}\right)\left(d + e^{ik_y}t_1\right) & \epsilon_c - 2d_1\cos k_x - 2t_2\cos k_y \end{pmatrix}, \tag{4}$$

where $\vec{k} = (k_x, k_y)$.

For $d_1 = t_1 = t_2 = t_0 = 0$, the Bloch Hamiltonian becomes the simplest case, i.e., 1D tunable diamond chain. Here, the intra-cell parameters and on-site energies in use are $d = 0.043, d_0 = 0.06$ and $\epsilon_a = \epsilon_b = \epsilon_c = 0.387$. For $d_1 = t_1 = t_2 = 0$ but $t_0 = d_0$, the 1D chains are connected via the side coupling, forming the 2D lattice. Band structures of the side-coupled 1D tunable diamond lattices are obtained as Fig. 2(b). One can quickly see that the band structures of the side-coupled tunable diamond lattices substantially differ from the 1D chain's band. However, these tight-binding band structures are still different from the band structures from the FDTD numerical simulations. Meanwhile, taking into account inter-cell hopping integrals ($d_1 = t_1 = t_2 = 0.002, t_0 = d_0$), as displayed in Fig. 2(c), we now show that the band structures are in good agreement with the numerical results, where the non-dispersive band is found to be in the third band, along $\Gamma \to X$ line only. Our FB modes are consequences of destructive interference — out-of-phase electric fields from the A and B sites cause destructive interference at the C site with the same coupling

strength through the spatial symmetry. One, therefore, fails to describe the photonic bands using the nearest-neighbor tight-binding consideration. The numerically calculated photonic bands are rather consequences of interactions through higher-order hoppings. Especially, the emergence of the compact localized states (CLSs) is secured even if the higher-order hopping interactions are taken into account for the tight-binding model.

III. FORMATION OF COMPACT LOCALIZED STATES FROM EXCITED MODES

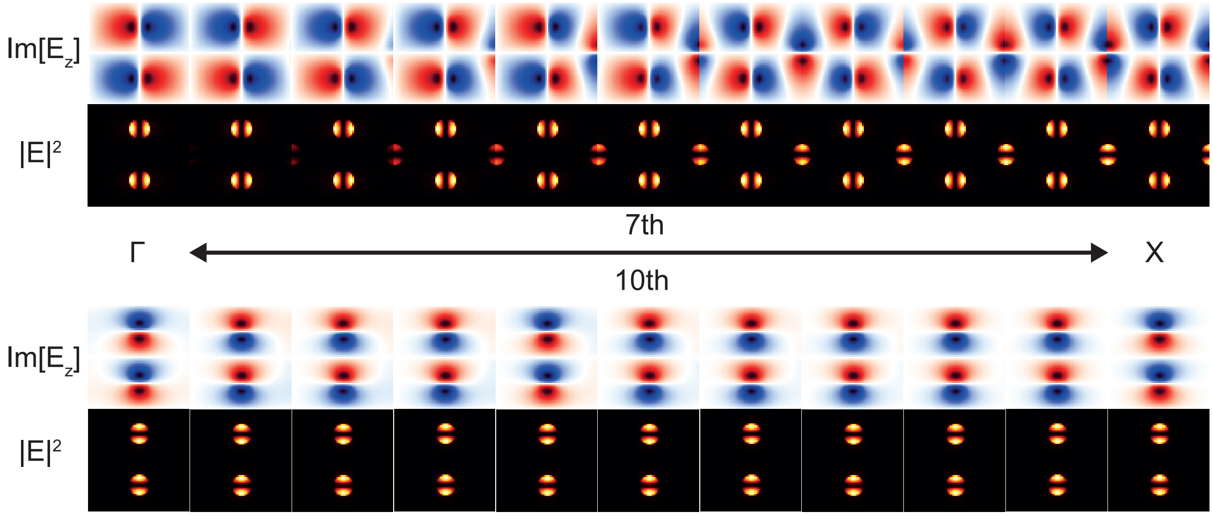

FIG. 3. Electric fields and field intensities of the 7th (upper pannels) and 10th (lower pannels) along the $\Gamma \to X$ line.

The formation of CLSs is a consequence of destructive interference at C site from out-of-phase electric fields from A and B sites. This mechanism is simply applied to the FB for fundamental modes. On the other hand, the CLSs are also formed as a result of destructive interference for excited modes, as we found the 10th band is non-dispersive. Electric field distribution for the 10th band shows electric dipoles in A and B sites with opposite phases to each other. However, the 7th band is also characterized by out-of-phase dipole distribution, but not flat along the $\Gamma \to X$ line. Figure 3 shows that the 7th band does not support the CLS formation with non-zero dipole distribution in C site, contrary to the 10th band.

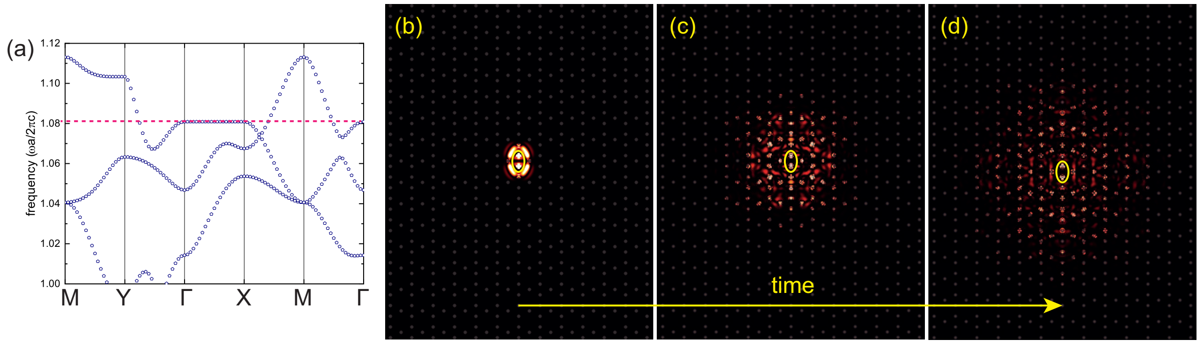

FIG. 4. (a) Photonic band structure of the 2D photonic crystal in the vicinity of the excite FB. The dashed line indicates the FB frequency. (b-d) Snapshots of field intensity distribution for different time steps with out-of-phase point sources for the FB frequency $f = 1.08088$.

IV. SELF-COLLIMATION EFFECTS BY THE EXCITED FB MODES

When the out-of-phase excitation is given for the fundamental FB frequency $f = 0.501107$, one observes the self-collimation effect due to the prohibited light propagation in x direction. On the other hand, the self-collimation effects are also expected to be for the excited FB frequency. However, Figure 4 shows that one cannot find the self-collimation effects even with out-of-phase excitation for the excited FB frequency $f = 1.08088$. The reason is the fact that at $f = 1.08808$, there are not only the dispersive modes propagating in y direction ($\Gamma \to Y$) but also dispersive modes in other directions like $X \to M$ and $M \to \Gamma$. Therefore, although we tune the frequency of the light source with the excited FB frequency very accurately, light propagation is not limited to the one direction, spreading out the photonic crystal.
